# Admission to Study Dentistry

**Published:** 1894-06

**Authors:** 


Article V.

### ADMISSION TO STUDY DENTISTRY.

There still exists a wide difference of opinion between the dental educationalists of England and the United States, as to the standard of education required from candidates for entrance to study. It would seem to be inferred, from many contributions in the journals, that, for reasons unknown or not declared, our American cousins, with a population of 60,000,000, with richly-endowed universities, a superior system of common-school education, a marvellous supply of literary and scientific institutions, magazines and papers, are not prepared to raise the matriculation to the standard required in Britain and Canada. The editor of the *International Journal*, in the March issue, remarks, "It is questionable whether the profession are ready to advance this beyond what is regarded as a good English education, and we are not sure that it would be advisable at the same present time, but it is an additional reform that must come. We can have no sympathy with the methods adopted in

England and on the Continent in this respect, and do not believe that the high standard there required can ever be adopted in this country, as far as dentistry is concerned. In order to meet its demands, a young man's best years are sacrificed to the attainment of information which, while in itself of great value, is utterly useless in a practical profession such as ours must ever remain. The change, if any be made, must be made to a slightly higher standard."

Why should not the ranks of dentistry be drawn from the higher educated class of the community? Why not from the universities in preference to the common school? It may be argued that some of the best men in our profession had a very limited education. It may also be stated, as a fact, that some of them had no education at all. But it will not be pretended that a low standard of education is a *sine qua non* of professional aptitude, or that a classical and mathematical education is a bar to success. If the preliminary examination at present demanded, would have shut out some of those who have honored our ranks for many years, could it not be argued that it is shutting out many to-day who might honor our ranks in years to come? Educationalists are not philanthropists. They should have no sentimental considerations. It is surely time, with all the educational advantages possessed, to demand a higher standard from young men who have to enter at once upon such studies as Anatomy, Physiology, Chemistry, Materia Medica, etc., ignorant of the very elements of the languages from which most of the terms in these sciences are derived. Such education is but a caricature and a travesty, and will explain much of the peculiar "scientific" discussions for which dentistry, more than any other profession, is distinguished. If educationalists mean to make dentistry nothing better than "practical," it will be in order for medical men to repossess the ground of scientific study and training, while dentistry proper descends again to the ranks of tooth carpentering. But if it is to be classed among the liberal and learned professions, as it certainly is in England and on the Continent, in Canada and Australia, it must at least aspire to exalt its standard of admission, so as to exclude those whose limited education hinders them from fully comprehending and assimilating scientific studies. It need be no barrier to an ignoramus, if the ignoramus determines to prepare himself for entrance. If he is incapable of such preparation, he is unfit to be better than an exclusive mechanic. Dentistry has long ago escaped from its purely mechanical probation, and it is time the reproach was removed that we are "fractionally qualified beings" whose science is mostly smatter.

Sixteen years ago Dr. Chales W. Elliott, President of Harvard University, delivered an address in Boston before the American Academy of Dental Science. We have frequently stated that we might quote eminent authorities in the United States to prove the weakness of American dental education, and it will perhaps suggest good grounds for advancement, by contrasting the situation to-day with what it was when Prof. Elliott spoke: "It is well known," he said, "that thousands of rude, ignorant men have entered the profession, attracted by its apparent profitableness and debarred by no law, to establish usage, and by no intelligent discrimination of the public against uneducated practitioners." . . . "As the future of a profession—whatever may be its present—is largely determined by the nature of the education which the youth who enter it receive, it is the condition of dental schools which should first engage the attention of those who wish to place dentistry on a level with the learned professions. All the evils which threaten the profession would gradually but surely disappear if dental schools could be made independent, strict and thorough, and public opinion could be so enlightened as to make the calling inaccessable or profitless to uneducated men." . . . "The first fact which strikes one, at the outset of an enquiry into the method and practices of dental schools, is that most of them do not demand, as a qualification for admission, any preliminary education whatever. No matter how ignorant and untrained a man may be, most dental schools are open to him. Until very recently all the medical and law schools in the United States were in the same ignominious condition. Among American professional schools, the theological schools alone, and not all of them, have escaped this degradation. It would be difficult to exaggerate the effect upon the estimation in which the profession of medicine and dentistry are held, of the fact that, until within two years, these professions have been accessible to men who could barely read and write, and have actually been entered by thousands of persons who never received, at school or college, the early training which, in the great majority of cases, is an essential preliminary to a life of refinement and cultivation."

It may not conduce to the numerical strength of college attendance that such advancement should be made, but there are not only far too many dental colleges, and too many students in most of them, but the educational standard of the large majority is not what it should be. It is very exceptional to find a student who has graduated in Arts disgracing dentistry by quack methods of advertising. It is very common to find a large proportion of the illiterate "Doctors of Dental Surgery" at the head of every unprofessional dodge, as they are, as a rule, at the tail end of any ethical or progressive reform. A high standard of matriculation would be the surest, even if it would be a slow antidode. The facilities for higher education in the United States are more democratic than in England.

Speaking of the preliminary examination in Arts required of all candidates for registration in England, Prof. Elliott adds: "There is no need of argument to prove that such conditions of entrance as these will, in the course of twenty years, greatly improve the quality of the mass of the profession in England and it is the mass, and not the few persons of exceptional gifts, that educational regulations are always intended to affect. If American dentistry, as a profession, is to maintain its rank in the world, it must be defended by similar requisitions against the incursion of inadequate men."—*Editorial in Dominion Dental Journal.*

